# Viral exanthema as manifestation of SARS-CoV-2 infection: A case report

**DOI:** 10.1097/MD.0000000000021810

**Published:** 2020-08-28

**Authors:** Gabriela Mariana Iancu, Adelaida Solomon, Victoria Birlutiu

**Affiliations:** a University Lucian Blaga of Sibiu, Faculty of Medicine, Dermatology Department; b Sibiu Emergency County Hospital, Clinic of Dermatology; c University Lucian Blaga of Sibiu, Faculty of Medicine; d Sibiu Emergency County Hospital, Clinic of Internal Medicine; e University Lucian Blaga of Sibiu, Faculty of Medicine, Infectious Diseases Department; f Sibiu Emergency County Hospital, Clinic of Infectious Diseases, Sibiu, Romania.

**Keywords:** exanthema, SARS-CoV-2, anosmia, clinical manifestations, immune response

## Abstract

**Rationale:**

The clinical manifestations of the SARS-CoV-2 infection are mainly respiratory but the virus can cause a variety of symptoms. Dermatological findings are less well-characterized. Data is scarce on their timing, type and correlation with the immune response.

**Patient concerns:**

We present the case of SARS-CoV-2 infection in a previously healthy woman who presented with respiratory symptoms and developed anosmia, diarrhea, and an erythematous maculo-papular rash on day 15 from symptom onset.

**Diagnosis:**

The nasopharyngeal swab tested by real time PCR for COVID-19 was positive. We interpreted this as a viral exanthema likely caused by an immune response to SARS-CoV-2 nucleotides.

**Interventions:**

She was treated with Hydroxychloroquine, Azithromycin and Lopinavir/Ritonavir, and the rash with topical corticosteroids.

**Outcomes:**

All symptoms resolved except for anosmia which persisted for 6 weeks. At the 4- and 6-weeks follow-up the IgG titers for SARS-CoV-2 were high.

**Lessons:**

We must consider that SARS-CoV-2 has a multi-organ tropism. In our case, the SARS-CoV-2 infection had lung, nasopharyngeal, neurological, digestive, and skin manifestations. Identifying the different manifestations is useful for understanding the extent of SARS-CoV-2 infection. We not only present a rare manifestation but also suggest that cutaneous manifestations may correlate with immunity.

## 1 Introduction

At the end of 2019, a novel coronavirus was identified as the cause of pneumonia cases in the city of Wuhan, China.^[1]^ The virus has spread to become a global pandemic.

Among the clinical features of the disease the most frequent manifestations are fever, cough, dyspnea, upper respiratory tract symptoms (rhinorrhea, sore throat), headache, myalgias, nausea, diarrhea, smell, and taste disorders confusion.^[2]^ All these are nonspecific and can be encountered in other viral respiratory infections but the development of pneumonia (dyspnea and bilateral infiltrates on lung imaging) several days after the onset of symptoms is highly suggestive in the present context.^[3]^

The course of the illness ranges from asymptomatic to severe. Some of the complications that have been described are: acute respiratory distress syndrome, acute cardiac injury, thromboembolism (pulmonary or stroke), cytokine release syndrome with elevated inflammatory markers (d-dimer, ferritin), Guillain-Barre syndrome, secondary infections.^[4]^ The recovery time is around 2 weeks for mild infections and between 3 and 6 weeks for severe disease.^[5]^

Various other symptoms have been associated with COVID-19 but the predictive value of a single symptom in the diagnosis is uncertain.

Dermatological findings have been reported (maculopapular rash, urticarial and chicken pox-like eruptions, transient livedo reticularis, chilblain lesions, etc.), but these are not well characterized.^[6]^ Pathogenetically, the appearance of cutaneous lesions during the SARS-CoV-2 infection can be explained by an immune response initiated by the viral nucleotides which activate Langerhans cells with the secondary involvement of keratinocytes (maculopapular, urticarial and chicken pox-like rashes), by microthrombi formation and cutaneous vasculopathy (chilblain lesions, livedo reticularis, erythema multiforme-like rash, gangrene), or by reaction to the medication administered (urticaria, erythroderma, erythema multiforme, etc.).^[7,8]^

We report a case of disseminated exanthema that appeared after 15 days of treatment for SARS-CoV-2 infection in a patient without other medical and dermatological problems in the past.

## 2 Case report

A 41 year old woman attended the Emergency Department for fever, myalgias, dysphagia, nasal congestion, headache, symptoms present for 2 days before presentation. She declares that she was in contact with a person who tested positive for SARS-CoV-2 a week before presentation. The patient is not a smoker and has no medical history of note.

On physical examination she was found to be of normal weight, with a temperature of 38.3°C, pharyngitis, billateral submandibular microlymphadenopathy, oxygen saturation of 98% in ambient air, blood pressure of 110/60 mm Hg, heart rate of 88/minute. The nasopharyngeal swab tested by rt-PCR for COVID -9 was positive and the patient was admitted.

The chest X-ray was unremarkable. The blood tests revealed an inflammatory syndrome: CRP 16.35 mg/L (RV 0-5), fibrinogen 494.7 mg/dl (RV 170-420), ESR 34 mm/hour (RV 0-20). The full blood count showed lymphopenia, with an absolute lymphocyte count of 1090/μl (RV 1500-4000) and a neutrophils to lymphocytes ratio of 2789. The D-dimers were slightly raised −603.32 ng/ml (RV 45-499). Other blood tests performed were normal (ferritin, LDH, procalcitonin, blood cultures, nasal and pharyngeal swabs, urine culture). The routine renal and liver tests were also normal. No abnormalities were noted on fundoscopy.

We initiated treatment (as per the national protocol) with Hydroxychloroquine 400 mg twice a day on the first day, then 200 mg twice a day until day 10, Azithromycin 500 mg/day for 5 days, with antifungal protection, and Lopinavir/Ritonavir 200/50 mg 2 tablets twice a day for 7 days. The patient became apyrexial on the second day of treatment.

**Figure 1:**
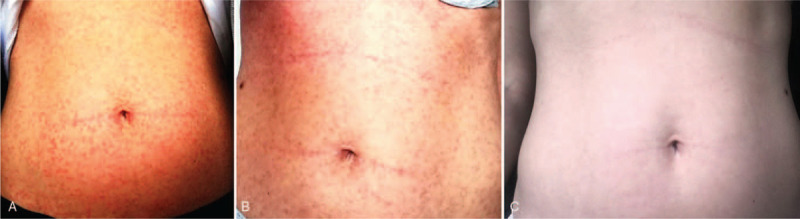
Disseminate exanthema due to SARS-CoV-2. A. Disseminate maculopapular purpuric-like rash, 1 day after onset. B. After 5 days the papule disappeared, the erythema faded, but the purpuric aspect was still present. Also, pink-brown maculae appeared. C. The aspect of the skin after 10 days of treatment for exanthema.

On the third day of treatment anosmia occurred (persisted for 6 weeks) and on the fourth day she reported dry cough, mild dyspnea, and diarrhea. Crepitations were heard on auscultation of both lung bases. The oxygen saturation was within normal limits (96%–98%). Systemic pulse corticosteroid therapy was given (methylprednisolone 120 mg intravenous daily for 3 days), under which the dyspnea and cough improved and no more rales are heard. The patient was discharged after a two-week hospital stay, following 2 negative SARS-CoV-2 tests (performed 24 hours apart). The diarrhea resolved 1 month after onset. One day after discharge an erythematous rash appeared initially on the trunk and disseminated over the next 5 days, centrifugally, to the proximal limbs. Dermatological examination describes a disseminated erythematous maculopapular rash, purpuric in appearance, mildly pruritic, with a tendency to confluency. The clinical appearance suggests a viral exanthema in the context of SARS-CoV-2 infection (Fig. 1A). Vitamin C and topical corticosteroids of medium potency were administered during the first days, followed by emollient, hydrating lotion thereafter. The rash extended centrifugally but spared the face, palms, and soles, as well as the mucous membranes. After 5 days of treatment the papule disappeared, the erythema improved but the purpuric appearance and mild pruritus persisted, along with the residual pink-brown macule (Fig. 1B). After 10 days, the exanthema disappeared almost completely, except for the persistence of discrete pink macule on the abdomen (Fig. 1C). Follow-up at 1 month showed complete resolution of the rash, no respiratory symptoms but persistence of anosmia. The sense of smell returned partially 6 weeks after onset of anosmia. Antibody testing for SARS-CoV-2 was undertaken at 4 and 6 weeks follow-up. IgM were undetectable but IgG were detectable - 106.87 AU/ml at 4 weeks and 117 AU/ml at 6 weeks (RV under 10 AU/ml). The patient donated plasma.

## 3 Discussions

The clinical manifestations of the SARS-CoV-2 infection are mainly respiratory, but also with multi-organ damage in variable proportions. In a study of 138 patients hospitalized in Wuhan the clinical features identified at onset were: 99% fever (not as common in other studies), 70% fatigue, 59% dry cough, 40% anorexia, 35% myalgias, 31% dyspnea, 27% sputum production.^[9]^ In a study of over 5000 patients in New York, only 31% had a temperature of over 38°C at presentation.^[10]^ Anosmia and dysgeusia have been identified as common symptoms in COVID-19. In a survey of 202 outpatients from Italy 64% reported alteration of smell or taste.^[11]^ Gastrointestinal symptoms (abdominal pain, nausea/vomiting, or diarrhea) were found in 18% of the patients in a systematic review.^[12]^

In our case, the initial symptoms were predominantly respiratory, then digestive (diarrhea), neurological (anosmia), without any other organ dysfunction. Mild lymphopenia was noted but the neutrophils to lymphocytes ratio of 2.789 was interpreted as a favorable prognostic factor. A neutrophils to lymphocytes ratio of over 3.3 is cited in literature as a negative prognostic factor.^[13]^ Using the online method suggested by Liang et al for calculating the risk of progression of SARS-CoV-2 infection towards severe disease, we obtained a moderate risk in our case.^[14]^

During the evolution of the case atypical cutaneous manifestations occurred, with rapid dissemination of an erythematous maculopapular, purpuric-like, mildly pruritic rash.

Exanthems are disseminated erythematous eruptions caused by medications (antibiotics, anticonvulsivants, tuberculostatics, antihypertensives, etc.), infections (respiratory viruses, enteroviruses, parvoviruses, herpesviruses, HIV, etc.), toxins released by some microorganisms (like staphylococcal scalded skin syndrome) or by autoimmune disease.^[15,16]^

Viral exanthems secondary to SARS-CoV-2 infection are rarely described in literature. The first dermatological manifestations described in the context of SARS-CoV-2 infection were chilblain lesions on the plantar extremities and livedo reticularis. During the last months more types of cutaneous lesions were described in infected patients: urticarial, chickenpox-like,^[17]^ vasculitic, morbilliform,^[18]^ etc. A meta-analysis of 19 studies published by Sachdeva et al, showed that cutaneous manifestations can precede respiratory ones by 3 days (12.50% of cases) or can appear after 13 days (69.40%) from the onset of respiratory manifestations in SARS-CoV-2.^[8]^ The medium interval for the appearance of cutaneous lesions was 9.2 days and the medium interval to remission was 8.7 days, according to a study of 132 COVID 19 positive patients with chilblain and erythema multiforme-like lesions, published by Fernandez-Nieto et al.^[19]^ In our case the exanthema appeared 15 days from the onset of symtpoms and remitted after 10 days. Although there was a procoagulation status in our case (slightly raised d-dimmers), there were no acro-ischemic or vasculitic lesions. The absence of vasculitic lesions in this case suggests the fact that the pathogenetic mecanism of the viral exanthema was an immune response to the viral nucleotides. We excluded a secondary drug reaction by the lack of “en cocarde” lesions, mucosal involvement and pruritus. In order to exclude other causes of viral exanthema we conducted serological tests (IgM for parvovirus B19, human herpesvirus-6, 7, and Epstein Barr virus) which were negative. The pharyngeal swab was negative for streptococcus and the mildly elevated inflammatory markers along with the absence of the specific enanthema effectively excluded scarlet fever. Particular about this case is the persistence of anosmia for 6 weeks after disease onset.

## 4 Conclusions

Aside from the respiratory tropism of the SARS-CoV-2 virus, which dominates the scientific interest at the moment, we must consider that SARS-CoV-2 has a multi-organ tropism. In our case the spectrum of manifestations included the respiratory, neurological, digestive, and skin lesions. Identifying the manifestations known so far is useful to better understand the real extent of SARS-CoV-2 infection. We not only present a rare manifestation but also suggest that cutaneous manifestations may correlate with immunity.

## Author contributions

Gabriela Mariana Iancu is responsible for the acquisition, analysis and interpretation of data, concept and design, investigation, methodology, resources, critically revising and validation the manuscript.

Adelaida Solomon is responsible for the acquisition, analysis and interpretation of data, investigation, methodology, resources and validation the manuscript.

Victoria Birlutiu is responsible for the concept, design, investigation, methodology, resources, critically revising and validation the manuscript.

All authors contribute to writing, reviewing & editing the original draft. All authors read and approved the final manuscript.
